# Gender and Ethnic Differences in Diabetes Self Care in Malaysia: An Individual Participant Meta-Analysis of Summary of Diabetes Self Care Activities Studies

**DOI:** 10.21315/mjms2023.30.1.14

**Published:** 2023-02-28

**Authors:** Cheong Lieng Teng, Verna Kar Mun Lee, Ganeson Malanashita, Lokman Hakim Sulaiman, Mohamad Adam Bujang

**Affiliations:** 1 Department of Family Medicine, International Medical University, Negeri Sembilan, Malaysia; 2 Department of Community Medicine, International Medical University, Kuala Lumpur, Malaysia; 3 Clinical Research Centre, Sarawak General Hospital, Sarawak, Malaysia

**Keywords:** adult, type 2 diabetes mellitus, Malaysia, meta-analysis, self care

## Abstract

**Background:**

Many published studies in Malaysia have examined and assessed self care among type 2 diabetes mellitus (T2DM) patients using the Summary of Diabetes Self Care Activities (SDSCA) scale. The current paper is a meta-analysis of related studies that also examines how gender and ethnicity influence and shape T2DM self care practices in Malaysia.

**Methods:**

We undertook a bibliographic search for studies conducted and published in Malaysia on T2DM adults using the SDSCA scale. This is a two-stage individual participant meta-analysis of SDSCA which synthesised the overall and subscale score based on gender and ethnic groups as well as the correlation between SDSCA and HbA1c.

**Results:**

We examined 11 studies that utilised SDSCA to analyse 3,720 T2DM patients. The overall SDSCA score was 33.46 (47.8% of the 7-day week). The subscale score for general diet, specific diet, exercise, blood glucose self-monitoring and foot care were 4.80, 4.09, 2.87, 1.80 and 3.21, respectively. A small but statistically significant better self care in some gender or ethnic groups was noted. The SDSCA diet subscale and HbA1c showed statistically significant correlation.

**Conclusion:**

The finding suggested Malaysian T2DM patients were deficient in exercise and blood glucose self-monitoring. In fact, overall self care among Malaysian adult T2DM patients appears to be suboptimal across gender and the three main ethnic groups. Greater efforts are therefore needed to educate Malaysian adult T2DM patients to improve their self care practices.

## Introduction

The latest national population survey in Malaysia revealed a diabetes prevalence of 18.3%. Currently there isn’t any large-scale survey of diabetes self care in Malaysia (1). Appropriate and consistent self-management is key to long-term health maintenance and complication reduction in chronic diseases such as diabetes mellitus (2). Diabetes self care is a multi-dimensional construct that can be measured using many rating scales. A systematic review by Lee et al. (3) identified 13 patient-reported outcome measures and the most commonly used tool was the Summary of Diabetes Self Care Activities (SDSCA) scale developed by Toobert et al. (4). In another systematic review of 27 studies by Mogre et al. (5), only six SDSCA studies from low-and middle-income countries were included and none of them was focused on Malaysians.

The SDSCA assesses respondents’ appropriate actions in five domains: i) diet; ii) exercise; iii) blood glucose testing; iv) foot care and v) smoking over the past 7 days. Bujang et al. (6) provided Malay translation of the SDSCA which proved to have adequate reliability and validity in the Malaysian context. Diabetes self care has been assessed frequently using SDSCA in Malaysia, with conflicting results among the three main ethnic groups: Malays, Chinese and Indians. Devarajooh and Chinna (7) for example, noted no difference but Siti Khuzaimah et al. (8) reported that Indians overall had better self care. Our literature search failed to identify any systematic review of Malaysian studies focusing on gender or ethnic differences in the SDSCA components of self care (diet, exercise, foot care, etc). This review was aimed at synthesising the overall SDSCA score and its subscales and compare them by gender and ethnicity.

## Methods

**Figure 1:**
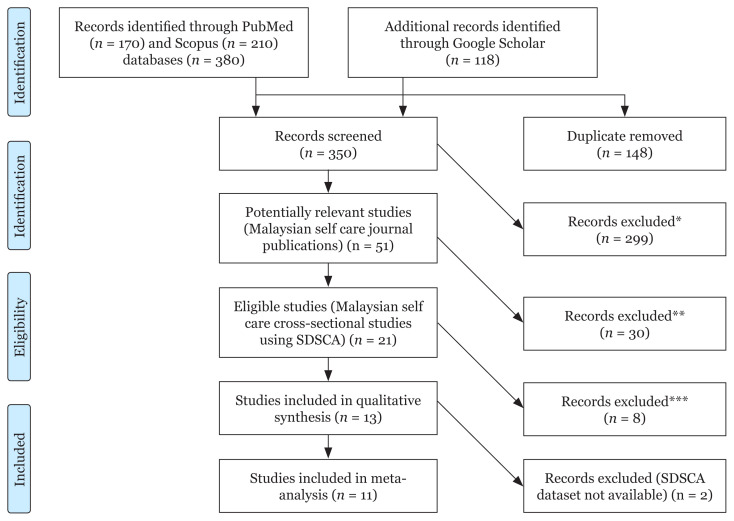
Flow chart showing search of studies Notes: *non-Malaysian studies = 43; non-journal publication = 28; reviews = 19; conference abstracts = 22; self care not measured = 81; non-diabetic studies = 22; not cross-sectional studies = 93 (some excluded publications are in more than one category); **SDSCA scale not used = 30; ***sample size < 100 = 3; studies using same datasets = 5

We searched PubMed (using the MESH terms ‘Self Care’, ‘Self-Management’ and ‘Diabetes Mellitus’ and ‘Malaysia’) and Scopus (using keywords ‘self care’ or ‘self management’ and ‘diabetes mellitus’ and ‘Malaysia’) from its inception until 31 Dec 2021. These were supplemented by Google Scholar search using the same keywords. The searching and processing of potentially relevant publications is as shown in Figure 1. These references were processed using Endnote 20 citation manager. Keywords of all references were coded based on publication types, study designs, study settings (primary care, hospital) and whether Summary of Diabetes Self Care Activity scale was used to measure self care.

The inclusion criteria were:

- cross-sectional studies conducted in Malaysia
- studies that used SDSCA scale
- study participants include at least 100 adult patients diagnosed with diabetes mellitus

Thirteen out of 21 studies fulfilled the above criteria. Corresponding authors of 11 studies supplied original SPSS datasets containing information on SDSCA and HbA1c. All datasets were merged and processed using IBM SPSS version 26.0 (Armonk, New York: IBM Corp). As many of the researchers did not record patients’ smoking status, this item was excluded in the meta-analysis. Thus, the SDSCA dataset consisted of 10 items (four items on diet, two items each on exercise, blood glucose self-monitoring and foot care) providing a total score between 0 and 70.

The following data were extracted from the manuscript and supplied datasets: number of study participants, study setting, language version of SDSCA, summarised demographic and glycaemic control data (age, gender, ethnicity and HbA1c) and scale reliability data (Cronbach α). The SPSS was used to generate mean, standard deviation (SD) and standard error of mean (SEM) of total and subscale SDSCA score of all participants, and selected prevalence data (see footnote of Table 2). Meta-analysis was performed using MedCalc Online Statistical Software version 20.006 (Ostend, Belgium: MedCalc Software Ltd); and for meta-analysis of studies with a continuous measure (comparison of means), MedCalc uses the Hedges g statistic as a formulation for the standardised mean difference under the fixed effects model. The heterogeneity statistic was incorporated to calculate the summary standardised mean difference under the random effects model. MedCalc uses the Hedges-Olkin method for calculating the weighted summary correlation coefficient under the fixed effects model, using a Fisher’s Z-transformation of the correlation coefficients. Next, the heterogeneity statistic was incorporated to calculate the summary correlation coefficient under the random effects model. We selected fixed effect model if the study heterogeneity (I^2^) was less than 50%, otherwise the random effect model was used.

The protocol of this systematic review was registered in INPLASY (9). This systematic reviewed was prepared following PRISMA guidelines (10, 11). The quality assessment of the studies was assessed using the Joanna Briggs Institute Critical Appraisal tools for use in JBI Systematic Reviews: Checklist for Prevalence Studies (12).

## Results

### Characteristics of SDSCA Studies from Malaysia

As described in Figure 1, we found 21 journal publications from Malaysia using SDSCA. Table 1 describes the characteristics of the 13 eligible studies. Out of these, 11 studies (8, 13–22) were included in the meta-analysis while two (7, 23) were excluded because the original datasets were not supplied by the authors (one corresponding author did not respond despite reminders; another declined to provide original SPSS dataset).

### Characteristics of SDSCA Studies Included in the Meta-Analysis

Eleven studies published between 2014 and 2020 provided SDSCA data for a total of 3720 adults diagnosed with T2DM. Table 2 summarises the SDSCA data at the whole scale, subscale, and at gender and ethnic group levels. Cronbach α of these studies varied between 0.614 and 0.741 (Table 1). There were some heterogeneities in the study settings and socio-demographic variables. The lack of clarity in the description of study participants and study setting contributed to low JBI scores in some studies (Table 1). The settings were either primary care clinics or hospitals (outpatient specialist clinics or wards) but in two studies, the specific study settings were not mentioned (15, 22). The study participants were mostly in their fifth decade; Ahmad Sharoni et al. (13) recruited only diabetes patient aged 60 years old and above. In terms of ethnic compositions, Ahmad Sharoni et al. (13) recruited mostly Malay participants but Papo et al. (19) who conducted his study in Sabah had very few Malay or Indian study participants due to the prevailing ethnic demographics there. Data on gender was available for all 11 studies but ethnic groups was missing in one study (17). Thus, the meta-analysis of ethnic group included only 10 studies.

### Meta-analysis of SDSCA Data

Meta-analysis of SDSCA score at the gender level did not detect any statistically significant difference (Table 3). However, a statistically significant difference was noted based on ethnic groups, where ethnic Indians had a higher SDSCA score compared with the Malay (standardized mean difference [SMD] = 0.144; 95% CI: 0.051, 0.236) and Chinese participants (SMD = 0.228; 95% CI: 0.109, 0.347) (Table 3).

Meta-analysis of SDSCA subscale score at the gender level revealed a statistically significant difference in exercise (SMD = 0.090; 95% CI: 0.025, 0.155) but not at all the other subscales (Table 4). A statistically significant difference was noted based on ethnic groups in these subscales: diet (better in Indians), exercise (worse in Malays), and foot care (worse in Chinese) (Table 4).

### Meta-analysis of Correlation between SDSCA and HbA1c

We generated Pearson’s correlation with SDSCA total score and diet scale score based on HbA1c data for eight studies (8, 13, 14, 16, 18–20, 22) (Table 5). The Pearson’s correlation between SDSCA total score and HbA1c varied between −0.498 and 0.126 while that between SDSCA diet subscale (general and specific diet) and HbA1c varied between −0.467 and 0.081. In view of the high level of heterogeneity, only the correlation between SDSCA diet subscale and HbA1c showed statistically significant correlation (pooled correlation = −0.123; 95% CI: −0.229, −0.014; *P* = 0.028).

## Discussion

The mean SDSCA score for all 3,720 study participants was 33.46. The mean SDSCA score based on gender and ethnic groups varied between 32.52 and 36.42. Since the maximum SDSCA score was 70, the mean score suggests that, on average, T2DM patients in Malaysia practised self care approximately 47.8% of the 7-day week which is considered relatively low. However, as stated by the developer of SDSCA, there is currently no specific cut-off level of SDSCA score that is considered as ‘good’ or ‘adherent’ (24). We are also unable to compare our summarised data with the review by Toobert et al. (4) due to absence of whole scale summarised data in the latter.

The mean score (and percentage of practice per week) for general diet, specific diet, exercise, blood glucose self-monitoring and foot care were 4.80 (69%), 4.09 (58%), 2.87 (41%), 1.80 (26%) and 3.21 (46%), respectively. The adherence to diet was reported to be slightly more than half of the week. It is uncertain whether this was due to over-reporting, as inaccuracy of self-reporting is well documented in Malaysia (25). Only 34.1% of T2DM patients performed adequate amount of exercise (i.e. at least 30 min at least five times per week). We were unable to find any Malaysian publication comparing SDSCA score and validated scale measuring physical activity (e.g. International Physical Activity Questionnaire [IPAQ]-7). Nor Shazwani et al. (26), in a cross-sectional study of T2DM patients in a Malaysian primary care clinic using IPAQ-7, reported a moderately high level physical activity of 66.7%. The performance of blood glucose self-monitoring was understandably very low as all the T2DM patients studied were seen in the public health facilities where the cost of glucometer and the test strips had to be borne by the patients (27). We found that 61.3% of all adult T2DM patients reportedly performed self-testing at least once a week; hence, it is possible that the ownership of glucometer among these patients could has increased since the last survey reported in 2007 where only 15.3% reported blood glucose self-monitoring (28). Since blood glucose self-monitoring among these patients can lead to better glycaemic control (29), greater effort is needed to promote the use of glucometer in the Malaysian public primary care clinics.

The meta-analysis of SDSCA total score and subscale score by gender and ethnic groups revealed statistically significantly higher score in certain domains, e.g. Indian fared better than Malays and Chinese in the domain of diet, females worse than male in exercise, Malays worse than Chinese or Indian in exercise, and Chinese worse than Malays or Indian in foot care. In the area of physical activity (measured using IPAQ), analysis of the National Health and Morbidity Survey data for 2011 supported the current finding, namely lower physical activity among female and Malays (30). However, a smaller study of physical activity (measured using IPAQ) among T2DM adults in one public primary care clinic surprisingly did not find higher physical activity among male patients (26).

There is some doubt whether the statistical differences observed above are clinically significant (as they represent less than 5% between-group differences). Previous Malaysian validation of SDSCA did not compare it with more objective outcome measures (e.g. diet record or IPAQ). However, as described in Table 5, the SDSCA datasets revealed negative linear correlation with HbA1c. Therefore, it is important to compare SDSCA with a more objective measure of self care practices in the local context.

This meta-analysis has the unique strength of synthesising individual participant data of one validated self care scale from one country. A possible weakness is that the self care data relied entirely on self-reporting, the accuracy of which may be somewhat contentious. Nonetheless, despite the limitation of SDSCA, adult T2DM patients in Malaysia notably have low practices of home blood glucose monitoring and exercise, necessitating specific intervention in the clinical setting. In view of the minor differences at the gender and ethnic level in self care, clinical intervention of diabetes self care in Malaysia may not need to be stratified based on these socio-demographic variables.

## Conclusion

The meta-analysis has shown that T2DM patients in Malaysia were deficient in exercise and blood glucose self-monitoring. Overall, their self care appears to be suboptimal across both gender and ethnic groups. Greater efforts, such as via educational programmes at the community and clinical levels, are needed to educate Malaysian adult T2DM patients on the importance of self care practices.

## Figures and Tables

**Table 1:** Characteristics of eligible studies

Study	N	Study site	Mean age (SD, range), year	Gender, male (%)	Ethnic groups (%)	SDSCA reliability	SDSCA data	HbA1c data	JBI score
Ahmad Sharoni et al. (13)	200	Hospital outpatient clinic	67.9 (5.7, 60–85)	59.5	M = 92.0, C = 7.0, I = 1.0	*n* = 200, Cronbach *α* = 0.763	mean = 27.22 (SD = 9.68)	*n* = 200, mean = 9.94 (SD = 1.64)	7
Chew et al. (14)	338	Primary care clinic	60.6 (10.1, 34–86)	44.3	M = 44.2, C = 32.6, I = 22.0, O = 1.2	*n* = 313, Cronbach *α* = 0.688	mean = 30.89 (SD = 12.37)	*n* = 305, mean = 8.41 (SD = 2.15)	8
Ching et al. (15)	151	Hospital, sites unclear	54.6 (12.9, 19–81)	66.2	M = 61.6, C = 11.9, I = 24.5, O = 2.0	*n* = 148, Cronbach *α* = 0.653	mean = 39.03 (SD = 12.03)	NA	4
Devarajooh et al. (7)	371	Primary care clinic	55.3 (10.1, NA)	38.0	NA	Dataset NA	Dataset NA	NA	Excluded
Jannoo et al. (16)	497	Hospitals, Primary care clinic	55.5 (11.0, 25–85)	53.7	M = 47.7, C = 17.5, I = 34.8	*n* = 497, Cronbach *α* = 0.688	mean = 34.37 (SD = 12.14)	*n* = 392, mean = 8.30 (SD = 2.87)	8
Kang et al. (17)	546	Primary care clinic	56.0 (11.6, 18–87)	51.5	NA	*n* = 546, Cronbach *α* = 0.741	mean = 34.11 (SD = 11.05)	NA	9
Kueh et al. (23)	200	Hospital outpatient clinic	NA	52.0	NA	Dataset NA	Dataset NA	NA	Excluded
Nur Khairul Bariyyah et al. (18)	536	Primary care clinic	56.6 (11.3, 17–92)	46.2	M = 55.9, C = 11.7, I = 31.3, O = 1.1	*n* = 450, Cronbach *α* = 0.709	mean = 34.69 (SD = 12.06)	*n* = 529, mean = 8.12 (SD = 1.84)	9
Papo et al. (19)	331	Primary care clinic	59.1 (11.4, 22–82)	47.4	M = 0.6, C = 12.1, I = 1.5, O = 85.8	*n* = 313, Cronbach *α* = 0.614	mean = 26.86 (SD = 11.11)	*n* = 331, mean = 7.57 (SD = 1.89)	8
Siti Khuzaimah et al. (8)	388	Hospital outpatient clinics	57.4 (10.7, 26–86)	57.5	M = 46.9, C = 18.0, I = 31.4, O = 3.6	*n* = 388, Cronbach *α* = 0.668	mean = 38.94 (SD = 11.93)	*n* = 388, mean = 8.32 (SD = 2.05)	9
Tharek et al. (20)	340	Primary care clinic	58.2 (12.1, 21–89)	41.2	M = 61.5, C = 19.4, I = 18.2, O = 0.9	*n* = 340, Cronbach *α* = 0.717	mean = 36.24 (SD = 11.68)	*n* =340, mean = 7.99 (SD = 1.71)	9
Tohid et al. (21)	360	Primary care clinic	53.4 (10.5, 27–80)	40.3	M = 72.2, C = 8.6, I = 17.8, O = 1.4	*n* = 320, Cronbach *α* = 0.642	mean = 31.05 (SD = 10.14)	Data unsuitable^a^	9
Yap et al. (22)	187	Multiple settings	52.5 (11.6, 23–81)	47.6	M = 49.2, C = 38.5, I = 11.8, O = 0.5	*n* = 187, Cronbach *α* = 0.709	m = 32.05 (SD = 11.38)	*n* = 143, mean = 8.69 (SD = 2.10)	5

Notes:

a not continuous data; Ethnic groups:

M = Malay, C = Chinese, I = Indian, O = Others; *N* = total sample size; *n* = sample size; NA = not available

**Table 2:** Total and subscale means for SDSCA based on combined SPSS dataset

Variables	N	SD	SEM	Mean (95% CI)
SDSCA whole scale (all participants)	3720	12.039	0.197	33.46 (33.07, 33.84)
Male only	1853	12.047	0.280	33.46 (32.91, 34.01)
Female only	1865	12.014	0.278	33.46 (32.92, 34.01)
Malay only	1612	11.833	0.295	33.44 (32.86, 34.02)
Chinese only	560	12.623	0.533	32.52 (31.48, 33.57)
Indian only	686	12.140	0.464	36.42 (35.51, 37.33)
SDSCA subscales				
Diet (general)	3835	1.910	0.031	4.80 (4.74, 4.86)
Diet (specific)	3826	1.421	0.023	4.09 (4.05, 4.14)
Diet (specific, eat five serving of fruits and vegetables)	3841	2.101	0.034	4.46 (4.39, 4.52)
Diet (specific, not eating high fat foods)	3842	2.045	0.033	3.73 (3.66, 3.79)
Diet (general and specific)	3807	1.336	0.022	4.45 (4.40, 4.49)
Exercise^a^	3840	2.122	0.034	2.87 (2.80, 2.94)
Blood glucose self-monitoring^b^	3805	2.114	0.034	1.80 (1.73, 1.87)
Foot care	3848	2.540	0.041	3.21 (3.13, 3.29)

Notes: CI = confidence interval; *N* = sample size; SD = standard deviation; SEM = standard error of mean;

a 34.1% of study participants reported exercising 30 min at least five times a week (based on SDSCA item 5; 1312/3855);

b 38.7% of study participants reported performing blood glucose self-monitoring zero time per week (based on SDSCA item 7; 1491/3856)

**Table 3:** Meta-analysis of total SDSCA scores by gender and ethnic groups

Comparison groups	*N*1	*N* 2	I^2^	SMD	SE	95% CI	t	*P*-value
Male versus female	1853	1868	0%	−0.028	0.033	−0.093, 0.037	−0.854	0.393
Malay versus Chinese	1612	560	16%	0.056	0.052	−0.046, 0.159	1.082	0.279
Indian versus Malay	686	1612	27%	0.144	0.047	0.051, 0.236	3.045	0.002
Indian versus Chinese	686	560	32%	0.228	0.061	0.109, 0.347	3.749	< 0.001

Notes: CI = confidence interval; N1 = sample size in first comparison group; N2 = sample size in second comparison group; SD = standard deviation; SEM = standard error of mean

**Table 4:** Meta-analysis subscale SDSCA scores by gender and ethnic groups

Subscale/comparison groups	*N*1	*N*2	I^2^	SMD	SE	95% CI	t	*P*-value
Diet (general and specific)								
Male versus female	1884	1805	54%	−0.052	0.050	−0.151, 0.046	−1.039	0.299
Malay versus Chinese	1663	566	70%	−0.059	0.101	−0.257, 0.138	−0.587	0.557
Indian versus Malay	714	1663	65%	0.268	0.088	0.095, 0.441	3.041	0.002
Indian versus Chinese	714	566	46%	0.207	0.060	0.089, 0.325	3.446	0.001
Exercise								
Male versus female	1901	1818	3%	0.090	0.033	0.025, 0.155	2.708	0.007
Chinese versus Malay	568	1687	0%	0.110	0.052	0.009, 0.211	2.139	0.033
Indian versus Malay	719	1687	0%	0.177	0.046	0.087, 0.268	3.850	< 0.001
Chinese versus Indian	568	719	0%	−0.105	0.060	−0.222, 0.013	−1.749	0.081
Blood glucose self-monitoring								
Male versus female	1890	1811	81%	0.092	0.077	−0.059, 0.243	1.196	0.232
Malay versus Chinese	1665	565	0%	0.079	0.052	−0.022, 0.181	1.529	0.126
Malay versus Indian	1665	711	2%	−0.024	0.046	−0.114, 0.067	−0.509	0.611
Chinese versus Indian	565	711	0%	−0.065	0.060	−0.182, 0.053	−1.081	0.280
Foot care								
Male versus female	1905	1815	56%	0.046	0.069	−0.089, 0.180	0.667	0.505
Malay versus Chinese	1694	567	70%	0.202	0.101	0.004, 0.400	2.001	0.046
Malay versus Indian	1694	722	59%	0.060	0.080	−0.098, 0.217	0.744	0.457
Indian versus Chinese	722	567	40%	0.181	0.060	0.063, 0.298	3.020	0.003

Notes: CI = confidence interval; *N*1 = sample size in first comparison group; *N*2 = sample size in second comparison group; SD = standard deviation; SEM = standard error of mean

**Table 5:** Correlation coefficient between SDSCA and HbA1c at study level and meta-analysis

Study	Correlation for SDSCA total score (95% CI)	*N*1	*P*-value	Correlation for SDSCA diet subscale (95% CI)	*N*2	*P*-value
Ahmad Sharoni et al. (13)	−0.498	200	< 0.001	−0.014	200	0.843
Chew et al. (14)	0.126	283	0.035	0.081	298	0.163
Jannoo et al. (16)	0.017	392	0.732	−0.010	392	0.850
Nur Khairul Bariyyah et al. (18)	0.046	442	0.335	−0.022	499	0.630
Papo et al. (19)	−0.100	331	0.069	−0.285	331	< 0.001
Siti Khuzaimah et al. (8)	0.020	388	0.699	−0.094	388	0.066
Tharek et al. (20)	−0.322	340	< 0.001	−0.177	340	0.001
Yap et al. (22)	−0.374	143	< 0.001	−0.467	143	< 0.001
Total		2519				
Meta-analysis, fixed effect (95% CI)	−0.095 (−0.134, −0.056)		< 0.001	−0.100 (−0.138 to −0.061)		< 0.001
Meta-analysis, random effect (95% CI)	−0.140 (−0.286, 0.012)		0.072	−0.123 (−0.229 to −0.014)		0.028
Heterogeneity (I^2^)	93%			87%		

Notes: CI = confidence interval; *N*1, *N*2 = sample sizes
